# Extubation Failure: A Case-Based Review of an Overlooked Airway Risk

**DOI:** 10.7759/cureus.84692

**Published:** 2025-05-23

**Authors:** Jennifer S Rackstraw, Lucille Roodt

**Affiliations:** 1 Department of Anaesthesiology, Chris Hani Baragwanath Academic Hospital, Johannesburg, ZAF

**Keywords:** airway risk stratification, difficult airway management, difficult extubation, extubation failure, extubation strategy, failed extubation protocol, high-risk extubation, postoperative airway complication, postoperative anaesthesia planning

## Abstract

Extubation is a critical yet often overlooked aspect of airway management. Although frequently performed in anaesthetic practice, extubation can be a complex and challenging event that remains under-represented in clinical teaching.

This report describes the case of a 41-year-old male patient who sustained facial and chest trauma following a pedestrian motor vehicle accident. He underwent open reduction and internal fixation of a mandibular fracture. Despite an uneventful induction with nasal intubation and intraoperative course, he experienced two episodes of extubation failure in theatre, both associated with hypoxia and hypercapnia, requiring urgent reintubation. Contributing factors included maxillofacial trauma, chest trauma, and likely airway oedema. Ultimately, he could not be safely extubated in theatre and was admitted intubated to an intensive care unit (ICU).

This case highlights the importance of structured, proactive planning for extubation in high-risk patients. It underscores the need for thorough risk stratification, anticipation of complications, and readiness to intervene in the event of extubation failure. It also draws attention to the need for greater emphasis on extubation management in anaesthetic training, as well as the potential role for validated tools to predict extubation risk.

## Introduction

Extubation is a pivotal step in airway management and carries a significant risk of complications, particularly in high-risk patients [1-5]. While difficult intubation is well recognised, studied and systematically addressed in anaesthetic teaching, extubation has traditionally received less attention [2,4]. Yet extubation failure can result in adverse outcomes, including hypoxia and cardiac arrest [1-5].

This report presents a case of extubation failure following maxillofacial surgery in a trauma patient and explores the differential diagnoses of extubation failure, evidence-based strategies for prevention and management, and the importance of structured extubation protocols. It also reflects on the broader implications for clinical practice and the need for research into predictive tools specific to extubation risk.

## Case presentation

​​A 41-year-old male patient with a history of paraplegia since birth presented for open reduction and internal fixation of a mandible symphysis fracture sustained one week earlier in a pedestrian vehicle accident. Additional injuries included multiple rib fractures with pulmonary contusions, bilateral pneumothoraces managed with intercostal drains, and a right scapula fracture managed conservatively.

His medical history was otherwise unremarkable. He had undergone an uneventful general anaesthetic in 2022 and had no known allergies or substance use.

Preoperatively, on respiratory exam, he had a left intercostal drain still in situ, with minimal swinging. Bilaterally, he had good air entry, and no wheezing or crackles were heard. He was tachypneic (respiratory rate of 30 breaths per minute) and maintained an oxygen saturation of 90% to 96% on room air. This was likely due to the lung contusions and resolving bilateral pneumothoraces. He was placed on supplemental oxygen via nasal cannula at 2 L/minute with oxygen saturation above 94%. He was in pain and was tachycardic with a heart rate of 112 beats per minute. Arterial blood gas (ABG) on room air showed a pH of 7.5, a partial pressure of oxygen (pO₂) of 79 mmHg, a partial pressure of carbon dioxide (pCO₂) of 30 mmHg, lactate of 0.8 mmol/L, HCO₃ of 23.4 mEq/L and a base excess of 0.2. On his airway examination, his jaw was fixated in place, allowing only an interincisor opening distance of 1 cm to 3 cm. He had a full range of motion of his neck, and his jaw protrusion could not be assessed. On systemic examination, the patient was alert and orientated. The cardiovascular examination was unremarkable.

In the operating theatre, standard American Society of Anaesthesiologists (ASA) monitoring was applied. Induction was achieved with 100 mg of propofol, 200 mcg of fentanyl, and, after successful bag-mask ventilation, 50 mg of rocuronium. Nasal intubation was successful on the first attempt using a direct laryngoscope with Macintosh blade 4 and Magill's forceps. The patient was given 4 mg of dexamethasone, 2 g of cefazolin, and a multimodal analgesia regimen (including 20 mg of ketamine, 1 g of paracetamol, and 6 mg of morphine). The surgery proceeded uneventfully.

At the end of the case, neuromuscular blockade was reversed with 2.5 mg of neostigmine and 0.4 mg of glycopyrrolate. This was done in accordance with institutional guidelines, as sugammadex supply is limited and withheld for emergency cases. Following extubation, the patient developed suspected laryngospasm, which was managed with 100% oxygen, jaw thrust, and positive pressure ventilation. During this episode, his vitals were as follows: a heart rate of 140 beats per minute and a blood pressure of 168/100 mmHg, and he desaturated to 50% for less than one minute until the laryngospasm resolved. Following this, adequate tidal volumes could still not be achieved with bag mask ventilation, and paradoxical breathing with bilateral crackles was noted. Differential diagnoses included negative pressure pulmonary oedema (for which 40 mg of furosemide was given) and upper airway obstruction (for which 8 mg of dexamethasone was given). There was a resolution of the paradoxical breathing and crackles.

Despite airway suctioning, a second episode of acute upper airway obstruction occurred, again managed with positive pressure ventilation. During this episode, his heart rate went up to 120 beats per minute, blood pressure was 150/90 mmHg, and oxygen saturation was 98%. Once able to mask ventilate him, the end-tidal carbon dioxide (CO₂) remained high at 80 mmHg. The patient was then reintubated using 200 mg of propofol and 50 mg of rocuronium. Oral intubation was successful on the first attempt using a direct laryngoscope with Macintosh blade 4. Laryngoscopy and fibreoptic bronchoscopy revealed no evidence of oedema, trauma, or obstruction. ABG showed a metabolic acidosis (pH: 7.28, PCO₂: 43 mmHg, PO₂: 146 mmHg on fraction of inspired oxygen (FiO₂): 70%, bicarbonate (HCO₃): 20.2 mEq/L, base excess: -6.5). The patient was adequately ventilated with a tidal volume of 8 ml/kg and peak pressure < 16 cmH2O in order to treat any possible underlying bronchoconstriction; 20 mg of ketamine and 200 mg of hydrocortisone were also administered.

Once stable, he was weaned to minimal FiO₂ and pressure support. Repeat ABG showed improvement (pH: 7.39, PCO₂: 33 mmHg, base excess: -4.2). He was then reversed with 200 mg of sugammadex, regained consciousness, and was following commands and achieving tidal volumes of 6 ml/kg with no pressure support. Unfortunately, no neuromuscular monitoring was available, and a train of four was not performed. A second extubation attempt was performed, but once again resulted in airway obstruction, requiring bag-mask ventilation. The patient was unable to maintain his own airway or adequate tidal volumes and required jaw thrust and positive pressure ventilation. During this episode, his heart rate was 130 beats per minute, blood pressure was 160/95 mmHg, oxygen saturation was 95%, and end-tidal CO₂ was 70 mmHg. He was reintubated (oral intubation) and admitted to the intensive care unit (ICU) for planned delayed extubation after 24-48 hours. The patient remained intubated in the ICU for 48 hours, after which he was extubated with no complications. At the time of discharge from the ICU, he was fully alert and orientated.

## Discussion

Extubation is an elective but critical step in airway management, and its associated risks are often underappreciated. Although the incidence of extubation failure in the operating room is relatively low (0.1% to 0.45%) [1-3], the consequences can be severe, including hypoxic brain injury and death [1-3]. Extubation is often performed when the patient’s physiology or anatomy has evolved, sometimes unfavourably, since intubation and the start of surgery [2]. This case highlights the importance of a structured approach to extubation in high-risk patients and the need to swiftly identify the causes of extubation failure when it occurs.

Differential diagnoses and diagnostic approaches

**Figure 1:**
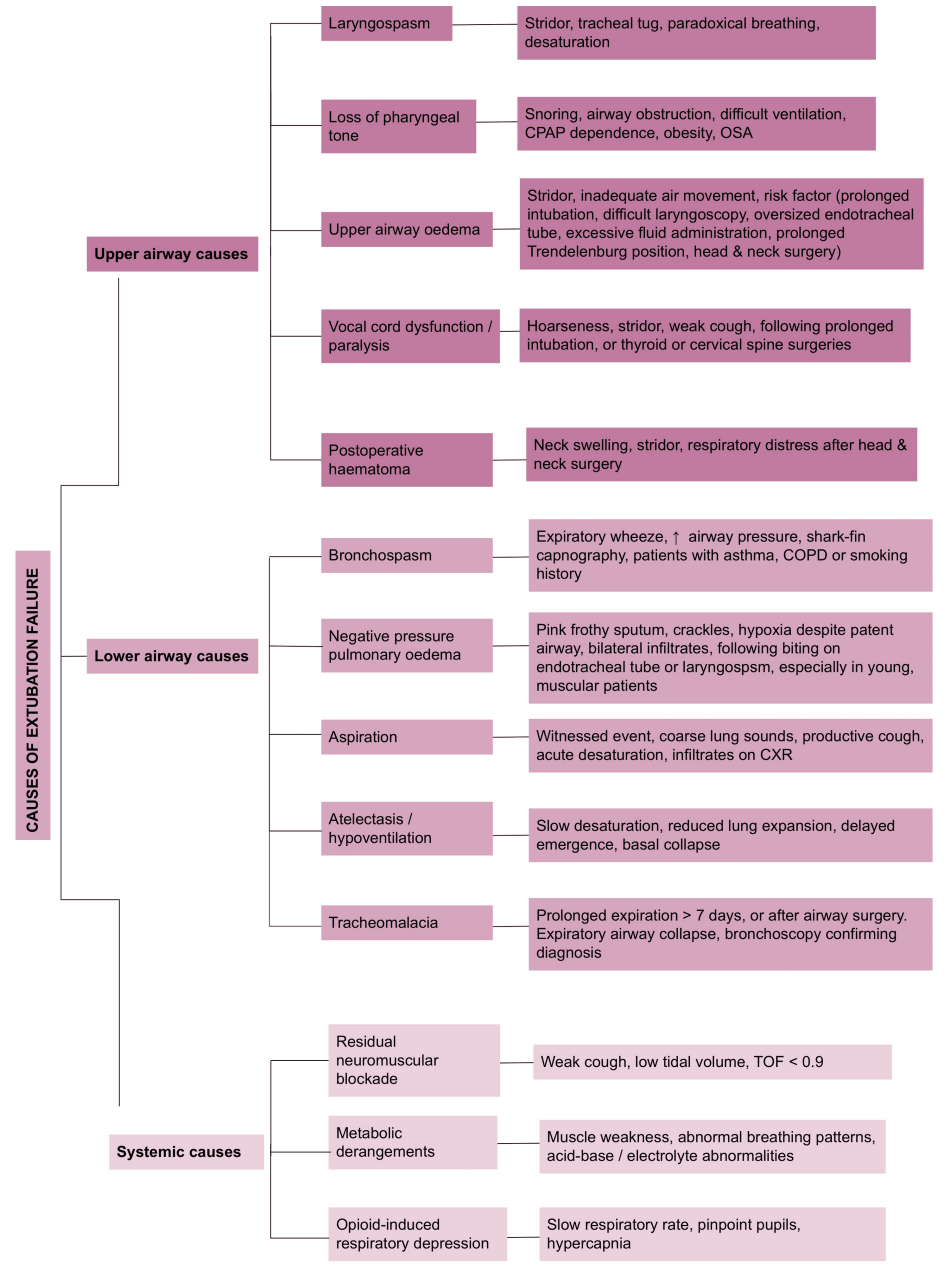
Causes of extubation failure Summary of potential causes of extubation failure with corresponding clinical clues [1-6]. CPAP: continuous positive airway pressure; OSA: obstructive sleep apnoea; COPD: chronic obstructive pulmonary disease; CXR: chest X-ray; TOF: train of four

A systematic, anatomical approach to differential diagnosis allows for rapid identification and management. See figure 1 for causes and diagnosis. Upper airway causes of obstruction typically present immediately after extubation. These include laryngospasm, vocal cord dysfunction, airway oedema, loss of pharyngeal tone, and postoperative haematomas. Laryngospasm, often triggered by blood, secretions or surgical stimulation [1-3], is a clinical diagnosis suggested by stridor, tracheal tug, paradoxical breathing and desaturation [2, 4]. Upper airway oedema may follow prolonged or difficult intubation [1,2], use of oversized endotracheal tubes [2], excessive fluid administration [3,5], Trendelenburg positioning [1,5], or head and neck surgery [1,2]. Its presence may be suspected based on risk factors, stridor, and inadequate air movement [1-3, 5] and confirmed via flexible bronchoscopy [3, 5]. Although the cuff leak test is sometimes used to predict oedema, its utility remains controversial in anaesthetics [1, 2, 4].

Vocal cord dysfunction or paralysis may result from prolonged intubation or thyroid or cervical spine surgery [1-3] and typically presents with hoarseness, stridor, and a weak cough [1, 2, 5]. Loss of pharyngeal tone is more common in patients with obesity, obstructive sleep apnoea, opioid use, or neuromuscular disease [5], suggested clinically by snoring, difficult ventilation, or continuous positive airway pressure (CPAP) dependence [5]. Postoperative haematomas, particularly after thyroid or neck surgery, must also be considered in the presence of neck swelling, stridor, and respiratory distress [2].

Lower airway causes of extubation failure typically result in progressive respiratory compromise. Negative pressure pulmonary oedema may develop following laryngospasm or biting on the endotracheal tube [3, 6], especially in young, muscular patients [4, 5]. It presents with pink frothy sputum, hypoxia despite a patent airway, and crackles on auscultation [3,4,6], supported by bilateral pulmonary oedema on chest X-ray and hypoxia on arterial blood gas [4-6]. Bronchospasm is more likely in patients with asthma, chronic obstructive pulmonary disease, or a smoking history [1, 2, 5] and is identified by expiratory wheeze, rising airway pressures, and a ‘shark fin’ capnography trace [1, 2, 5].

Aspiration should be suspected after witnessed events, acute desaturation, coarse lung sounds, and a productive cough [2, 5]. Chest X-ray may show dependent infiltrates [2, 5], and flexible bronchoscopy can confirm aspirated contents [5]. Tracheomalacia, more common after prolonged intubation (>7 days) [1, 2] or following trauma and airway surgery [3, 6], may cause expiratory airway collapse, confirmed bronchoscopically [2, 6]. Atelectasis and hypoventilation are commonly due to residual anaesthesia, high opioid use, obesity, or pain and result in impaired oxygenation and ventilation [5]. These are suggested by reduced lung expansion, slow desaturation, and delayed emergence, with arterial blood gases and chest X-rays aiding diagnosis [5].

Systemic causes must also be considered, including residual neuromuscular blockade [1, 6], opioid-induced respiratory depression [1, 5], and metabolic disturbances [5]. Residual neuromuscular blockade is a commonly considered cause, especially in the setting of inadequate reversal and in the setting of renal or hepatic impairment [1, 6]. Signs include a weak cough, low tidal volumes, and a train-of-four ratio <0.9 [5, 6]. Opioid-induced respiratory depression can be suggested by slow, shallow breathing, pinpoint pupils, and hypercapnia [5]. Although data from a large quality assurance database suggests that incomplete reversal and opioid-induced respiratory depression are actually relatively uncommon causes of immediate postoperative reintubation [6], Metabolic derangements, including acidosis and electrolyte abnormalities, can weaken respiratory effort and should be confirmed by blood testing [5].

Planning and risk stratification

Extubation should never be considered routine, especially in high-risk patients. A structured, risk-based approach is critical, as advocated by the Difficult Airway Society (DAS) [2], the Canadian Airway Focus Group (CAFG) [7], and the All India Difficult Airway Association (AIDAA) [8]. These guidelines recommend categorising patients as low, intermediate, or high risk based on both airway-specific and systemic factors [2]. Airway risk factors include a known or anticipated difficult intubation, restricted airway access, head and neck surgery, anticipated oedema or bleeding, or risk of aspiration [2, 4]. General risk factors include obesity, significant respiratory or cardiac disease, neuromuscular weakness, electrolyte or acid-base imbalance, abnormal body temperature, and haemodynamic instability [2, 4, 7].

In this case, the combination of facial trauma, bilateral lung injury, and maxillofacial surgery would classify the patient as high risk, warranting a proactive extubation strategy or planned delayed extubation.

After risk stratification has occurred, the next step involves addressing all modifiable risk factors. Airway patency should be evaluated using direct or indirect laryngoscopy to exclude oedema, haematomas, clots, or foreign bodies [1, 2]. The cuff leak test may be used to assess subglottic oedema [1,2,7], although its limitations must be acknowledged [4,8]. Lower airway factors such as trauma, infection, oedema, or secretions should also be considered [2].

Neuromuscular blockade must be fully reversed, ideally with quantitative monitoring to ensure a train of four ratio ≥0.9 [2, 3, 6, 7]. Other physiological factors, including temperature, acid-base status and electrolyte balance, should be corrected, and analgesia optimised [2].

A retrospective study by Brueckmann et al. [9] proposed a scoring system to predict postoperative respiratory complications. This includes ASA class ≥3 (three points), emergency surgery (three points), high-risk procedure (two points), history of congestive cardiac failure (two points), and chronic pulmonary disease (one point) [9]. Patients with scores ≥7 had a reintubation rate of 6.4%, compared to 0.1% in those with a score of 0 [9]. Such tools may help guide perioperative planning. 

When the risk of extubation failure is high and modifiable risk factors cannot be corrected, extubation should be delayed. Ongoing ventilation in a high-dependency setting allows time for resolution of physiological or airway pathology and may reduce the likelihood of failed extubation and associated morbidity [2, 7].

Execution of extubation 

Once risk factors have been addressed, extubation should be deliberate and well-planned. Extubation must occur in a controlled environment ('sterile cockpit') with minimal distractions, skilled personnel and access to difficult airway equipment [2, 7]. In high-risk cases, extubation should be planned with a multidisciplinary team, such as senior anaesthetists or ENT specialists, particularly when there is a risk of 'cannot intubate, cannot oxygenate' [2,7]. Preoxygenation with an FiO₂ >0.9 is recommended to increase oxygen reserve [1, 2, 4, 7]. No universal extubation position is recommended, but reverse Trendelenburg may benefit obese patients, while the left lateral position may reduce aspiration risk [2, 4]. Suctioning should be done under direct vision, especially where blood is present [2]. Alveolar recruitment manoeuvres may reduce atelectasis, although evidence for postoperative benefit is limited [1, 2].

The endotracheal tube should be deflated and removed at end-inspiration to promote immediate exhalation and minimise aspiration risk [2, 7]. Bite blocks, such as oropharyngeal airways or gauze rolls, should be used to prevent occlusion and negative pressure pulmonary oedema [2, 4]. Awake or deep extubation strategies may be employed based on patient factors, although awake extubation is likely to be safer in high-risk cases [2].

Adjuncts and rescue strategies

In high-risk extubations where reintubation may be difficult, the use of an airway exchange catheter (AEC) is a valuable adjunct. It allows for continued airway access after extubation and facilitates rapid reintubation if necessary [2, 3, 7, 10].

In the case of post-extubation upper airway obstruction or stridor, immediate management includes high-flow oxygen, basic airway manoeuvres, continuous positive airway pressure, and nebulised adrenaline [1, 2]. If airway oedema is suspected, corticosteroids, such as 100 mg of hydrocortisone six hourly, may be beneficial, provided they are administered for at least 12 hours pre-extubation. A single pre-extubation dose is not considered effective [2].

Post-extubation monitoring and support

Vigilant post-extubation care is essential, with postoperative oxygen supplementation, as well as close clinical monitoring, as a normal oxygen saturation may mask hypoventilation [2]. Patients with airway compromise should receive high-flow humidified oxygen, ideally with end-tidal CO₂ monitoring [2]. Patients should be nursed upright [2]. Deep breathing and coughing should be encouraged, and CPAP or nasopharyngeal airways should be used where indicated, particularly in patients with obstructive sleep apnoea [2].

Lessons learnt

This case reinforces several important clinical lessons. Firstly, even when induction and intraoperative management are uneventful, extubation may still present significant challenges. This patient had multiple risk factors, including preexisting paraplegia, facial trauma, bilateral chest injury and maxillofacial surgery, as well as preoperative tachypnoea with lower oxygen saturation, which should have prompted a high-risk extubation strategy or even a delayed extubation. Although extubation was appropriately performed with neuromuscular reversal and suctioning, recurrent hypoventilation and obstruction suggest that the underlying pathology may not have been fully optimised.

There were, however, aspects of care that were well managed. There was a prompt response to signs of airway obstruction with appropriate recognition, ventilation, and reintubation. The use of fibreoptic bronchoscopy also likely minimised further harm.

In hindsight, further measures such as delaying extubation, using an AEC, or planning for postoperative non-invasive ventilation may have further reduced the risk of failure. This case highlights the importance of ongoing risk assessment, multidisciplinary planning, and structured extubation protocols, as well as appropriate postoperative placement for high-risk extubation.

Extubation is not simply the reversal of intubation but must be approached with the same care and planning, particularly in patients with multiple risk factors. Structured guidelines, risk assessment tools, and further research are needed to improve outcomes. While general postoperative respiratory scoring systems exist [9], extubation-specific predictive tools are still lacking.

This case underscores the importance of reporting and analysing extubation failures to refine practice, improve protocols, and ultimately enhance patient safety in anaesthetic care.

## Conclusions

In conclusion, this case highlights the complexities of managing extubation in patients with multiple risk factors. Despite careful planning, unforeseen challenges can arise. This case reinforces that extubation is not merely the reversal of intubation but often occurs under compromised physiological conditions. It emphasises that extubation is a critical, elective process requiring thorough preparation, risk stratification and ongoing reassessment.

The case also underscores that vigilant post-extubation monitoring is essential to detect and address airway and ventilatory complications early. Finally, it highlights the need for better predictive tools specific to extubation failure and continued improvement of practical guidelines for high-risk extubations in order to enhance safety and outcomes in anaesthetic practice.
